# Nutritional and Health Benefits of Jackfruit (*Artocarpus heterophyllus* Lam.): A Review

**DOI:** 10.1155/2019/4327183

**Published:** 2019-01-06

**Authors:** R. A. S. N. Ranasinghe, S. D. T. Maduwanthi, R. A. U. J. Marapana

**Affiliations:** Department of Food Science and Technology, University of Sri Jayewardenepura, Gangodawila, Nugegoda, Sri Lanka

## Abstract

*Artocarpus heterophyllus* Lam., which is commonly known as jackfruit is a tropical climacteric fruit, belonging to Moraceae family, is native to Western Ghats of India and common in Asia, Africa, and some regions in South America. It is known to be the largest edible fruit in the world. Jackfruit is rich in nutrients including carbohydrates, proteins, vitamins, minerals, and phytochemicals. Both the seeds and the flesh of jackfruit are consumed as curries and boiled forms, while the flesh in fully ripen stage can be eaten directly as a fruit. Several countries have developed different food products such as jam, jellies, marmalades, and ice creams using pureed jackfruit. The several parts of jack tree including fruits, leaves, and barks have been extensively used in traditional medicine due to its anticarcinogenic, antimicrobial, antifungal, anti-inflammatory, wound healing, and hypoglycemic effects. Despite all these benefits, unfortunately, the fruit is underutilized in commercial scale processing in regions where it is grown. The aim of this review is to disseminate the knowledge on nutritional and health benefits of jackfruit, in order to promote utilization of jackfruit for commercial scale food production.

## 1. Introduction

Jackfruit is one of the commonly consumed foods in Sri Lanka from the ancient time. It is a nonseasonal fruit and had a major contribution to the food supply of the people and their livestock when there were short supplies of staple food grains [1, 2]. Therefore, it is referred to as poorman's food [3].

It is a monoecious tree and both male and female inflorescences are found on the same tree [4, 5]. The fertilization is by cross-pollination and the propagation is mostly through seeds. The complete fruit development process takes about three to seven months from the pollination, varying in different countries. [6].

### 1.1. Origin and Distribution

Jackfruit is considered to be originated in the rain forests of the Western Ghats in the Southwestern part of India, but some authors argue that Malaysia could be the possible centre of origin [7]. It is found in many parts of Asia, Africa, and South America [2, 6, 8, 9]. Jacktree grows in warm and moist regions [4, 10].

### 1.2. Jacktree and the Fruits

Jacktree is a medium-sized evergreen tree, and typically reaches 8-25 m in height [11]. The tree grows rapidly in early years, up to 1.5 m/year (5 ft/year) in height, slowing to about 0.5 m/year (20 in/year) as the tree reaches maturity [12]. It has a straight rough stem and a green or black bark which has a thickness of around 1.25cm, exuding milky latex [13].

The leaves are broad, elliptic, dark green in colour and alternate. They are often deeply lobed when juvenile on young shoots. Male heads are usually sessile or on short peduncles receptacles and sometimes born on the ultimate twing, while female heads are oblong ovoid receptacle [11–14].

Jackfruit has a relatively high productivity, about 25.71 t/ha [15]. The fruits are borne in the main and side branches of the tree [16]. A mature jacktree can yield from ten to two hundred fruits [17–21].

They are dicotyledonous compound fruits [22], which are oblong cylindrical in shape, and the length of the fruits ranges from 22 to 90 centimeters with the diameter 13-50 centimeters. The weight of individual fruits may vary between 2 and 20 kilo grams, and larger fruits of about 50 kilograms have been recorded [20, 23, 24].

Jackfruit has a green to yellow brown exterior rind that is composed of hexagonal, bluntly conical carpel apices that cover a thick, rubbery, and whitish to yellowish wall [11]. It is a multiple aggregate fruit which is formed by the fusion of multiple flowers in an inflorescence [16]. About 30% of the fruit weight is occupied by the flesh [8, 9, 20, 23, 24]. There are large number of bulbs inside the fruit, which have high nutritional value.

The fruit is made up of three main regions. They are

1. the fruit axis;
2. the persistent perianth;
3. the true fruit.

Due to the presence of laticiferous cells that produce latex, which helps to hold the fruits together, the axis and the core of the fruit are inedible.

The perianth is made up of three regions:

1. the bulb (the lower fleshy edible region);
2. the middle-fused region that forms the rind of the syncarp;
3. horny nonedible region commonly known as the spikes [11, 22].

The fruit colour changes from yellowish green to yellow due to the conversion of chlorophylls, anthocyanins, and carotenoids like pigments during ripening [25]. Depending on the variety, the colour of the bulb can be cream, white, light yellow, yellow, deep yellow, lemon yellow light saffron, saffron, deep saffron, or orange [8].

Jackfruit seeds are light brown, rounded, 2-3 cm in length by 1-1.5 cm in diameter [11]. They are surrounded by the flesh and enclosed in a white aril surrounding a thin brown spermoderm, which covers the fleshy white cotyledon. It has been found that these are rich in carbohydrates and proteins [16, 26].

**Figure 1:**
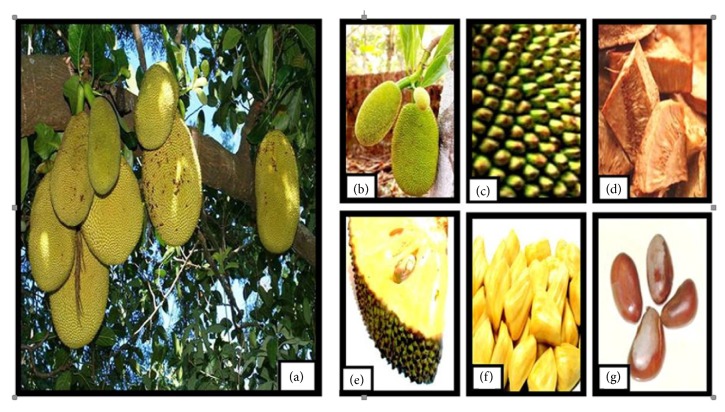
(a) Jackfruit tree with the fruits of different sizes; (b) jackfruit tree with the fruits in different stages of fruiting; (c) the jackfruit with conical carpel apices; (d) raw jackfruit pieces used for curries; (e) the interior of a ripe jackfruit with the seed; (f) the ripe eatable flesh of jackfruit; and (g) the jackfruit seed (source: [6]).

Jackfruits in different maturity stages and jackfruit seeds are shown in the Figure 1.

There is a widespread belief that excessive consumption of jackfruit flakes can lead to certain digestive disorders. The utilization of jackfruit as a commercial crop is limited due to its wide variations in fruit quality and long seed dormancy [21].

The fruits must be utilized as early as possible when it reached the maturity as very sharp off flavours can be developed. Therefore, it is practiced to harvest the fruit when it is firm and in a semiripen condition before ripening on the tree and then store until becoming soft and fit for processing [25].

### 1.3. Varieties of Jackfruit

Several studies including Hossain [27]; Saha* et al*. [28]; and Jagadeesh* et al*. [8] have reported diversity in jackfruit, based mainly on morphological, phenotypic, and organoleptic characteristics like the size of the tree, structure of the leaf, fruit form, age of fruit bearing, quality of the fruit flesh, their size, shape, density of spines, colour, texture, odor, quality, and period of maturity [18, 29]. According to Singh [30] and Vinning and Moody [31], there are at least 30 strains of jackfruit in the Indian subcontinent and 30 more types in Malaysia. In Sri Lanka, several jackfruit cultivars such as ‘Vela', ‘Varaka (Waraka)', ‘Peniwaraka', ‘Kuruwaraka', Singapore, or the Ceylon Jack are distributed [6].

However, there are two main varieties of jackfruit: firm and soft. In the firm variety, the perianth remains firm even at full ripeness, while in the soft variety the perianths become soft and fleshy on ripening [2]. The soft variety has fruits with small, fibrous, soft, and spongy flakes with very sweet carpels, whereas the firm variety is crunchy with crisp carpels and not sweet as the soft variety. The firm variety is considered to be of high quality [14]. Some studies have reported variations in the starch, total sugar, and reducing sugar contents of soft and firm types [2, 32].

### 1.4. Chemical Composition and Nutritional Value

The chemical composition of jackfruit varies depending on the variety. When compared with other tropical fruits jackfruit flesh and seeds contain more protein, calcium, iron, and Thiamine [18, 33, 34].

A study has explored that the ripe jackfruit is richer than apple, apricot, avocado, and banana in some minerals and vitamins [25].

The caloric content of jackfruit is low, where 100 g of jackfruit only contains 94 calories [35].

Table 1 depicts the composition of jackfruit according to the findings of several studies [18, 19, 36–39].

Several studies have found that there is a variation in chemical composition of jackfruit in different maturity stages [25].

### 1.5. Carbohydrates

Rahman* et al*. [40] have reported the presence of a high percentage of starch in jackfruit perianth and seed according to various chemical and histological studies. The starch and dietary fiber content of the flesh increase with the maturity [2].

According to a study carried out by Chrips* et al*. [41], the carbohydrate concentration of different varieties of jackfruit seed may vary from 37.4% to 42.5%.

### 1.6. Proteins

Jackfruit contains amino acids like arginine, cystine, histidine, leucine, lysine, methionine, threonine, and tryptophan [42]. The flesh of ripe jackfruit contains 1.9 g proteins per 100g. The protein concentration of the jackfruit seeds may vary from 5.3 to 6.8% [41]. According to Goswami* et al*. [43], the protein content of the flesh of different varieties of ripen jackfruit has ranged from 0.57 to 0.97%.

### 1.7. Vitamins

Jackfruit is rich in vitamin C [44]. Moreover, it is one of the rare fruits that is rich in B-complex group of vitamins and contains very good amounts of vitamin B6 (pyridoxine), niacin, riboflavin, and folic acid [16].

The changes of vitamin contents in different maturity stages of jackfruit have been evaluated by Tiwari and Vidyarthi [25]. The amounts of vitamins according to the study are presented in the Table 2.

### 1.8. Minerals

Samaddar [21] has recorded that the flakes of ripe jackfruits are high in nutritive value. Further he stated that every 100 g of ripe flakes contains 287-323 mg potassium, 30.0-73.2 mg calcium, and 11-19 g carbohydrates. The ripen jackfruit contains minerals such as calcium, magnesium and vitamins, and organic acids [25].

The mineral composition of the edible jackfruit flesh at different maturity levels is shown in Table 3, as reported in Tiwari and Vidyarthi [25].

### 1.9. Fiber Content

A study has found the fiber content of jackfruit to be 0.33-0.40% with no significant changes in different portions of the fruit at different ripening stages [45]. In Coronel [46], it has been reported that the fiber content of immature and ripe jackfruit is 2.6% and 0.8%, respectively. Another study has found slight variations in fiber content among the jackfruit fleshes in different varieties, ranging from 0.50 to 0.90 % [43]. Rahman* et al*. [2] indicated that the total dietary fiber of the perianths was almost similar in soft and firm varieties, but in Hasan [47], the fiber content of the jackfruit flesh varied from 0.57 to 0.86%, depending on the variety and the season.

### 1.10. Phytochemical Content in Jackfruit

Studies including Arung* et al*. [48]; Chandrika* et al*. [49]; Lin and Lu [50]; Ong* et al*. [45]; Venkataraman [51]; and Wong* et al*. [52] have shown that jackfruit contains many classes of phytochemicals such as carotenoids, flavonoids, volatile acids sterols, and tannins, with varying concentrations depending on the variety. According to Wongsa and Zamaluddien [53], total phenolic content in jackfruit is 0.36 mg GAE/g DW (milligrams of gallic acid equivalent per gram of dry weight).

Carotenoids are a class of natural pigments present in plants, animals, algae, and microorganisms which impart yellow-reddish colours. In addition to their colourant properties, they have provitamin A activity and are known to have beneficial effects on several chronic degenerative diseases, such as cancer, inflammation, cardiovascular disease, cataract, and age-related macular degeneration [10, 54–57]. The jackfruit kernel is reported to contain *β*-carotene, *α*-carotene, *β*-zeacarotene, *α*-zeacarotene and *β*-carotene-5,6*α*-epoxide, and a dicarboxylic carotenoid and crocetin [49], according to recent studies the key carotenoids present in jackfruit are all-trans-lutein, all-trans-*β*-carotene, all-trans-neoxanthin, 9-cis-neoxanthin, and 9-cis-vio-laxanthin [6].

Singh [30] has observed that the carotene content of jackfruit increased gradually with the progress of ripening.

Table 4 demonstrates the concentrations of different types of carotenoids present in jackfruit according to a study conducted by Faria* et al*. [10].

Wong* et al*. [52] have found 32 novel volatile components in jackfruit. According to the study, the esters, which impart the desired flavour to the fruit, are found in high concentrations in jackfruit.

### 1.11. Maturity Signs and Harvesting of Jackfruit

According to Palipane and Rolle [58] and Ramli [59], the maturity indices of jackfruit are as follows:

1. The fruit matures in about 12-16 weeks after flower anthesis.
2. Fruit colour changes from green to green yellowish.
3. A dull, hollow sound is produced when the fruit is tapped by the finger.
4. Fruit spinel becomes well developed and wide spaced.
5. The last leaf of the peduncle yellows.
6. An aromatic odor develops.
7. The number of spikes on the outer skin is decreased and spikes become flatter.
8. Fruit length and girt are increased.

Fruit should be harvested by cutting from the stalk using sharp bladed equipment. If the fruit is high up in the tree, a sack should be tied around the fruit with a rope, the stalk should be cut, and the fruit should be gently lowered to the ground [58].

Accurate determination of maturity and best harvesting time and correct harvesting practices allows minimum loss of fruits [25].

### 1.12. Uses of Jacktree

Different maturity stages of jackfruit flesh are consumed fresh or as canned slices, fruit juice, and dried chips. Fully ripen stage produce fruit juice of good eating quality with suitable aroma, texture, sweetness, and taste [1]. In some countries, pureed jackfruit has been processed into baby food, juice, jam, jelly, base for cordials, candies, fruit-rolls, marmalades, jackfruit leather, and ice cream [16, 60, 61]. The unripe stage is also used to prepare pickles, when the fruit is tender [25]. The seeds are also consumed after boiling and roasting or added to flour for baking and cooked in dishes [14]. Jacktree is used for its durable timber, which acquire reddish orange colour when aging. The timber also has antitermite properties [14] and used for the preparation of furniture [6]. The chips are used to extract an orange-red dye, which is used to colour the robes of Buddhist monks. The leaves and fruit wastes of the jacktree are used produce fodder for cattle, pigs, and goats [29]. Many parts of the plant, including the bark, roots, leaves, and fruits, are known for their medicinal properties in traditional and folk medicine [6, 62, 63].

### 1.13. Beneficial Effects of Jackfruit on Human Health

“Jackfruit (*Artocarpus heterophyllus* Lam) is a rich source of several high-value compounds with potential beneficial physiological activities” [64]. It is well known for its antibacterial, antifungal, antidiabetic, anti-inflammatory, and antioxidant activities [65].

Elevation of blood LDL: HDL ratio is one of the major risk factors for the development of coronary heart diseases [66]. Oxidation of LDL contributes to atherosclerosis which involves a series of inflammatory and oxidative modifications within the arterial wall [67, 68]. Free radicals also promote tissue injury, protein oxidation, DNA damage, and induce proinflammatory responses [69].

Antioxidants are the compounds that are able to delay, retard or prevent oxidation process [70]. They protect the body and biomolecules from the damage caused by generation of excess free radicals. Jackfruit contains a wide range of phytonutrients such as carotenoids that can act as antioxidants [6, 16]. Jagtap* et al*. [64] state that the antioxidant activities of jackfruit flesh extracts are correlated with the total phenolic and flavonoids content. According to Soong and Barlow [71], fresh seed and flesh possess substantial ascorbic acid equivalent antioxidant effects and 27.7 and 0.9 gallic acid equivalent phenolic contents, which are believed to have contributed to about 70% of the total antioxidant activity.

Jackfruit contains functional compounds that have capability to reduce various diseases such as high blood pressure, heart diseases, strokes, and bone loss. It is also capable of improving muscle and nerve function, reducing homocysteine levels in the blood [44].

Jackfruit is also rich in potassium which aids in lowering blood pressure and reversing the effects of sodium that causes a rise in blood pressure that affects the heart and blood vessels. This in turn prevents heart disease, strokes and bone loss and improves muscle and nerve function [44]. Vitamin B6 present in jackfruit helps to reduce homocysteine levels in the blood, consequently lowering the risk of heart disease [72].

Jackfruit is also a good source of vitamin C, which protects the skin from the damage that occurs as a consequence of the natural aging process and prolonged exposure to sun [73]. Vitamin C is also essential for the production of collagen, gives firmness and strength to the skin [74], and maintains oral health.

Some studies have also reported the anti-inflammatory effects of isolated bioactive compounds from the fruits of* Artocarpus heterophyllus* [75]. Jackfruit contains flavonoids which are effective in inhibiting the release of inflammatory mediators from the mast cells, neutrophils, and macrophages [76].

Phytonutrients such as lignans, isoflavones, and saponins in jackfruit contribute to its anticancer, antihypertensive, antiulcer, and antiaging properties. They prevent the formation of cancer cells in the body and fight against stomach ulcers [6]. The results of a study carried out by Ruiz-Montanez* et al*. [77] suggested that the jackfruit possesses compounds with chemoprotective properties to reduce the mutagenicity of aflatoxin B1 (AFB1) and proliferation of cancer cells and the jackfruit flesh contains compounds that may be an effective aid to prevent or treat lymphoma cancer.

Niacin in jackfruit is necessary for energy metabolism, nerve function, and the synthesis of certain hormones [16].

Dietary fiber present in jackfruit makes it a good bulk laxative. This decreases the exposure time and binds to cancer causing chemicals, as well as mineral and vitamins in the colon, and helps to protect the colon mucous membrane [5]. High fiber content also maintains smooth bowel movements and prevents constipation [16].

The flesh and seeds of jackfruit are considered as a cooling and nutritious tonic [16].

Jackfruit has abundance of important minerals [36]. It is rich in magnesium, which is important for the absorption of calcium and helps strengthen the bones and prevents bone-related disorders such as osteoporosis. Iron in jackfruit helps to prevent anemia and aids in proper blood circulation and copper plays an important role in thyroid gland metabolism [26].

According to Prakash* et al*. [11] and Rama Rao and Venkataraman [78], jackfruit possesses compounds like morin, dihydromorin, cynomacurin, artocarpin, isoartocarpin, cyloartocarpin, artocarpesin, oxydihydroartocarpesin, artocarpetin, norartocarpetin, cycloartinone, betulinic acid, artocarpanone, and heterophylol which are useful in fever, boils, wounds, skin diseases, convulsions, diuretic, constipation, ophthalmic disorders, snake bite, etc.

Jackfruit is also known for its antifungal properties. Trindade* et al*. [79] found a chitin-binding lectin named jackin, which has the ability to inhibit the growth of* Fusarium moniliforme* and* Saccharomyces cerevisiae*. It also exhibited hemagglutination activity against human and rabbit erythrocytes [79].

According to a study carried out by Fernando* et al*. [72], the hot water extract of jackfruit leaves significantly improved glucose tolerance in the normal subjects and the diabetic patients when investigated at oral doses equivalent to 20 g/kg.

Methanolic extracts of the stem and root, barks, heart-wood, leaves, fruits, and seeds of jackfruit have exhibited a broad spectrum of antibacterial activity [80]. Nematicidal activity against various nematodes including* Rotylenchulus reniformis*,* Tylenchorhynchus brassicae*,* Tylenchus filifofmis,* and* Meloidogyne incognita *also has been revealed by the jackfruit shoots [81].

Jackfruit wood extract is also known for inhibition of melanin biosynthesis. Some prenylated, flavones-based polyphenols, isolated from the jackfruit wood, have been shown to inhibit in vivo melanin biosynthesis in B16 melanoma cells, with little or no cytotoxicity [48].

Due to all these numerous health benefits the consumption of jackfruit flesh has increased in recent years [77].

### 1.14. Reasons for Underutilization of Jackfruit

Despite its nutritional values and enormous health benefits, the jackfruit is underutilized and not classified as a commercial crop and is rarely grown on regular plantation scale due to its short shelf life and insufficient processing facilities in the regions where they are grown [20].

About 60% of the whole jackfruit consists of inedible parts such as outer prickly rind, inner perigones, and central core [82] and only around 35% of the whole fruit consist of edible flesh [19].

The jackfruit flesh is highly perishable and often undergoes flavour loss, tissue softening, and cut surface browning [83]. The softening of the fruit makes it more susceptible for bruising and mechanical injury [59]. In ripe fruits, the spoilage is commonly observed in localized pockets of the large fruit [19, 84]. Following the harvesting, large quantities of ripe jackfruits undergo rapid deterioration due to lack of proper knowledge on postharvest practices which result in poor handling and inadequacy of sanitary practices and storage facilities in areas where they are processed and marketed [83, 85].

Due to its high perishability, jackfruits are usually exported as whole fruits and more than half of the fruit consists of inedible waste materials, which make it less cost effective. The inconsistency of the size and shape of the fruit make the design of packaging very complicated and rough and thick skin and the latex makes difficulties in preparation [59]. Jackfruit is a large fruit and the peeling is rather difficult [86]. Also, the separation of jackfruit edible bulbs from jackfruit rind is a difficult and more labour intensive task [87] and consumes more time, which makes it unattractive to urban population where most of them have a busy life style. Also, the intense flavour of the fresh fruit makes it unacceptable to some consumers [87] and there is a widespread belief that excessive consumption of jackfruit can lead to problems of digestion [6].

Also, the vast variations in the physical properties and biochemical compositions of fruits in different plant types make it difficult for the use of jackfruits from different trees for variety of products [87].

The jackfruit seeds contain around 55% moisture content, thereby lessening the possibility of being kept for a long time [88]. Usually jackfruit seeds are consumed in boiled or roasted form, but most of the jackfruit seeds are discarded [89].

In jackfruit processing industries, a huge amount of inedible parts such as peel are generated as waste, and usually they are used as animal feed. However, a limited number of researches have been devoted for the investigation of possibilities for conversion of these wastes into value-added products. Hence, a significant amount of jackfruit waste is discarded [90], creating serious waste disposal and environmental problems [91].

Therefore, advanced processing technologies and sustainable waste management strategies should be considered when processing jackfruits in commercial scale.

#### 1.14.1. Solutions to Increase the Use of Jackfruit and Research and Developments Based on Increasing the Utilization of Jackfruit

Introduction of high yielding jackfruit varieties, adhering to proper harvesting and postharvest practices such as appropriate handling, transportation and storage, development of novel processing technologies, and searching for new applications to minimize postharvest and production losses as well as conversion of jackfruit waste into value-added products would be better options for popularizing the jackfruit cultivation and consumption along with waste management of jackfruit processing industries.

Harvesting of jackfruit in green mature stage can prevent the mechanical damage. Also, adaption to appropriate postharvest practices may facilitate the exportation through extended shelf life. Storage of whole jackfruit at 10°C and 85-90% humidity can extend the shelf life of the crop approximately by two weeks [59].

At present, there is a growing consumer demand for wholesome, nutritional, and convenient food products. Therefore, in recent years, there is a growing market for minimally processed fruits and thereby increasing the concern for minimizing the damage to the fruits through correct handling and storage methods [86]. Processing of jackfruit into value-added products such as precut or ready to eat bulbs may remove the difficulty in separating the bulbs from the rind and conserve time and thereby may commercialize it among the urban population. It also enhances the crop's potential both locally and internationally through reducing the packaging and transportation costs, maintaining the quality and the freshness, and minimizing the quarantine barriers in some importing countries [59, 92]. However, once the fruit is cut, it can undergo rapid deterioration due to the physiological stress caused by physical damage [93]. Hence, appropriate techniques for the minimization of the quality loss should be investigated. The Modified Atmospheric Packaging along with low temperature storage can successfully extend the shelf life of minimally processed jackfruit [92]. Vargas-Torres* et al*. [86] have discovered the ability of pretreatment with 1-methylcyclopropene and the application of edible coatings (xanthan, sodium alginate, or gellan) to extend the shelf life of precut jackfruit up to 12 days, while preserving the original quality attributes when stored at 5°C. The findings of the study revealed that the pretreatment and application of edible coating were able to reduce the weight loss, respiration, and ripening rates while maintaining the desired sensory and nutritional attributes such as colour, firmness, pH, total soluble solids, and titratable acidity of the products. Saxena* et al*. [84] have tested for the effects of calcium treatment, osmo-blanching and drying methods on physicochemical and sensory attributes of jackfruit slices. The study revealed that optimization of pretreatment conditions together with combination of freeze-drying and hot-air drying can result in jackfruit bulb slices with better sensory attributes, discovering the possibility for commercial scale processing. Also, the dehydrated jackfruit bulbs in its powder form can be incorporated into other food products [84]. According to Ramli [59] there was a slower texture loss in calcium treated jackfruit bulbs, compared to untreated bulbs, and they had a shelf life up to 14 days when stored at 8°C.

Production of salty snacks such as chips from jackfruit flesh may attract the consumers for consumption of jackfruit as it may impart variety to their diet. Jagadeesh* et al*. [87] have conducted a study with the aim of determining the ideal physicochemical quality parameters for chips purpose jackfruit. The study revealed that the dry matter content, starch content, total soluble solids, and reducing sugar content have a major influence on the yield and quality of the product. The morphological characteristics and physical parameters such as thickness and size of jackfruit bulbs have a greater impact on the appearance, suitability for processing, and uniformity of frying operations. Hence, selecting the jackfruit genotypes with suitable characteristics is crucial for the sustainability of the production [87].

Mondal* et al*. [83] have taken an attempt for the development of products such as jam, jelly, pickle, and squash by processing different parts of jackfruit and to assess their nutritional quality. It was found that the products including jam, jelly, and squash could retain desirable quality up to six months and started to deteriorate after 8-9 months, while the quality of pickles remained unchanged even after 12 months of storage.

Fermentation of surplus or over-ripe jackfruit for the production of wine would be an interesting alternative for the effective utilization of the fruit. Jackfruit wine is reported to possess good antioxidant properties and protective effects against radiation induced DNA damage [85].

Furthermore, advanced processing technologies including freeze-drying, vacuum frying and cryogenic freezing have been used to develop new jackfruit-based food products [19, 59].

In recent years, the interest towards the utilization of alternative sources of starch in industrial applications is being increased. Hence, many researchers have paid their attention on jackfruit seeds as a potent source of starch [88]. Jackfruit seeds contains a considerable amount of starch, which is around 20% (dry basis) and as revealed by Tulyathan* et al*., [94], the recovery yield of starch extracted from jackfruit seeds was about 77%, which implies its possibility of being used as a potent source of starch in food and pharmaceutical industries [95], as a stabilizer, thickener, and a binding agent [96]. Conversion of fresh seeds into flour can be considered as an effective way of enhancing its utilization [88].

There are few recent studies which have emphasized the properties and utilization of jackfruit seed starch. According to Kittipongpatana and Kittipongpatana [95], the amylose content of jackfruit starch is around 24-32%, which is similar to potato starch. However, Tran* et al*. [97] have reported a higher amylose content (~44%) in jackfruit seed starch, which were cultivated in Vietnam.

The water and oil absorption capacities of jackfruit seed flour have found to be 200% and 90%, respectively, which provide its desirable functional properties for food industrial applications [88]. The gelatinization temperature of jackfruit seed starch was higher compared to starch from other sources [95], and the stability of jackfruit seed starch granules against thermal and mechanical shear has found to be high, enabling its application in products that require starch with such properties [35]. Santos* et al*. [98] have found jackfruit starch to be acid-resistance in solution and paste form.

The modification of jackfruit seed starch by physical and chemical means results in modified jackfruit starches with altered or improved properties including gelatinization temperature, water solubility, solution viscosity, swelling ability, and water uptake as well as resistance to enzymatic degradation [95]. Dutta* et al*. [99] have conducted a study on the effect of different extraction conditions on properties of acid-alcohol modified jackfruit seed starch, which can have possible applications in confectioneries, paper, and textile industries. The study revealed that jackfruit starch gels possess high degree of freeze stability.

The possibility of incorporating jackfruit seed starch as a thickener and stabilizer in chili sauce has been investigated by Rengsutthi and Charoenrein [96]. The ability of jackfruit seed starch to maintain the pH, titratable acidity, and total soluble solids in chili sauce has been found when incorporated in 1% level. As revealed by the study, jackfruit seed starch incorporated chili sauce has exhibited a superior quality when compared to chili sauce which was incorporated with corn starch. Cereals bars with good sensory and nutritional properties have been developed by the incorporation of 11% jackfruit seed flour and 27% of dehydrated jackfruit pulp [100].

Jackfruit seed also contains a significant amount of nonreducing sugars which makes it suitable to be utilized as prebiotics [101]. Several studies have successfully utilized jackfruit seed as a source of carbon for the extracellular production of pullulan by* Aureobasidium pullulans* MTCC2195 [102] and polyhydroxybutyrate using* Bacillus sphaericus *NCIM 5149 [103]. Nair* et al*. [104] have utilized jackfruit seed powder as a substrate for the production of L-lactic acid using* Streptococcus equinus* with the aim of value addition of an agro waste material by biotechnological intervention.

The possibility of utilization of jackfruit seed starch powder as a novel natural superdisintegrant in irbesartan fast dissolving tablets has been investigated by [105]. Nagala* et al*. [106] have extracted oils with essential fatty acids and antioxidant activities from the seeds of five different jackfruit varieties.

Jackfruit seeds also have been tested for its potent applications as a raw material for ethanol production, which can be considered as a renewable source of energy [107] and as a protein source in food industry due to its high protein content [108].

The outer peel of jackfruit is rich in fibrous compounds, calcium, and pectin and utilization of jackfruit peel for the production of pectin can contribute to the economic development of the regions where they are grown by creating more income for farmers and processors as well as reducing the environmental impacts of waste [90]. Begum* et al*. [109] have conducted a study to determine the impact of different extraction conditions on the yield and physicochemical and structural properties of pectin derived from jackfruit waste. The extracted pectin was of low quality with regard to its poor solubility and high ash content, compared to commercial pectin, raising the need for more advanced researches focusing on production of high-quality pectin from jackfruit waste with improved solubility. Hence, several studies have focused on extraction of pectin from jackfruit peel by the use of more advanced techniques including ultrasound assisted extraction [90] and ultrasonic-microwave assisted extraction [82], which were able to result in higher pectin yield compared to conventional methods. Govindaraj* et al*. [110] have revealed the possible utilization of jackfruit peel derived pectin in bone healing applications.

Jackfruit peel also has been tested for its suitability to be used as an efficient raw precursor for the production of activated carbon using different techniques such as phosphoric acid activation [91] and microwave induced NaOH activation [111], with the purpose of increasing the economic value, reduction of the cost of waste disposal and providing a potentially inexpensive raw material for commercial scale activated carbon production which in turn prevent deforestation since the utilization of wood for activated carbon production can be minimized [91]. Also, jackfruit peel can be used for the production of biooil, which can be used as an alternative for nonrenewable fossil fuel resources [112].

Ashok* et al*. [113] have conducted a study on development of a natural photo-sensitizer from jackfruit rags for dye sensitized solar cells, with the aim of exploring ways for upcycling the waste materials for energy harvesting.

Renuka Prasad and Virupaksha [114] have purified a protease named ‘artocarpin', from jackfruit latex which has showed proteolytic activity against casein. The jackfruit latex also can be used as an adhesive [59].

A study focusing on the investigation of nutritional quality of jackfruit stalk has been conducted by the Department of Food Science and Technology, University of Sri Jayewardenepura, Sri Lanka (unpublished data).

All these research efforts exhibit the possibility of utilization of jackfruit in commercial scale, with the aim of promoting its consumption.

## 2. Conclusion

Jackfruit is a tropical tree, which is a rich source of nutrients such as carbohydrates, proteins, vitamins, minerals, dietary fiber, and phytochemicals. Previous studies have revealed numerous health benefits of jackfruit including anticarcinogenic, antimicrobial, antifungal, anti-inflammatory, wound healing, and hypoglycemic properties. However, it is considered as an underutilized fruit in commercial scale, mainly due to higher percentage of inedible portion which leads to more waste generation, difficulty in peeling and separation of edible bulbs from the rind, lack of knowledge on proper postharvest practices, and inadequate processing facilities in regions where they are grown. Hence, adhering to correct postharvest practices and conversion of jackfruit into minimally processed products, such as precut jackfruit, may encourage more population towards the consumption of jackfruit, and conversion of jackfruit waste materials into valuable products may aid in waste management. There are only a few recent studies that have focused on the extending shelf life of jackfruit and value addition of jackfruit waste by converting them to different products and renewable energy sources. Thus, more researches should be devoted for discovering possible industrial applications of jackfruit and proper management of waste generated in jackfruit processing.

## Figures and Tables

**Table 1:** Composition of jackfruit (100 g edible portion).

**Composition**	**Young fruit**	**Ripe fruit**
Water (g)	76.2 - 85.2	72.0 - 94.0
Protein (g)	2.0 - 2.6	1.2 - 1.9
Fat (g)	0.1 - 0.6	0.1 - 0.4
Carbohydrate (g)	9.4 - 11.5	16.0 - 25.4
Fibre (g)	2.6 - 3.6	1.0 - 1.5
Total sugars (g)	-	20.6
Total minerals (g)	0.9	0.87 - 0.9
Calcium (mg)	30.0 - 73.2	20.0 - 37.0
Magnesium (mg)	-	27.0
Phosphorus (mg)	20.0 - 57.2	38.0 - 41.0
Potassium (mg)	287-323	191-407
Sodium (mg)	3.0-35.0	2.0-41.0
Iron (mg)	0.4-1.9	0.5-1.1
Vitamin A (IU)	30	175-540
Thiamine (mg)	0.05-0.15	0.03-0.09
Riboflavin (mg)	0.05-0.2	0.05-0.4
Vitamin C (mg)	12.0-14.0	7.0-10.0
Energy (KJ)	50-210	88-410

Sources: [18, 19, 36–39].

**Table 2:** Changes in vitamins in jackfruit flesh.

**Age of the fruit (in days)**	**Vitamin content in flesh (mg/100g)**		
	**B1**	**B2**	**C**
45	3.9	35.7	18.5
55	14.2	124.2	19.67
65	12.6	122.7	23.1
75	Trace	133	24.03
85	trace	48.2	22.5

Source: [25].

**Table 3:** Mineral composition of the edible fruit flesh at different maturity levels.

**Age of the fruit (in days)**	**Macro element (mg/100g)**		**Micro element ** **(mg/100g)**							
	**Ca**	**Mg**	**Cd**	**Co**	**Cr**	**Cu**	**Fe**	**Mn**	**Ni**	**Pb**
45	28.4	37.8	0.0	0.0	-	0.28	4.24	0.56	-	0.08
55	29.86	37.38	0.0	0.0	-	0.26	2.64	0.56	-	0.32
65	26.9	36.92	0.0	0.02	-	0.36	1.20	0.54	-	0.28
75	33.8	36.52	0.0	0.0	-	0.30	1.84	0.56	-	0.28
85	31.28	36.96	0.0	0.0	-	0.38	3.26	0.56	-	0.20

Source: [25].

**Table 4:** Concentration (*μ*g/100 g fresh weight) of carotenoids of jackfruit and their vitamin A values (*μ*g RAE/100 g fresh weight).

**Carotenoids **	**Concentration (** ***μ*** **g/100 g fresh weight)**
All-trans-neoxanthin	8.85
9-cis-Neoxanthin	6.87
All-trans-neochrome	0.88
All-trans-luteoxanthin	2.06
cis-Antheraxanthin	1.12
9-cis-Violaxanthin	7.05
cis-Luteoxanthin	0.34
All-trans-lutein	37.02
All-trans-zeaxanthin	0.96
All-trans-zeinoxanthin	1.72
cis-Zeinoxanthin	0.90
All-trans-*α*-cryptoxanthin	0.35
All-trans-*β*-cryptoxanthin	1.21
15-cis-*β*-Carotene	0.18
13-cis-*β*-Carotene	2.45
All-trans-*α*-carotene	1.24
All-trans-*β*-carotene	29.55
9-cis-*β*-Carotene	0.79
Total carotenoids	107.98
Vitamin A value	2.84

(Source: [10])

## Data Availability

The numerical data supporting this review article are from previously reported studies and datasets, which have been cited, and are available from the corresponding author upon request.
